# A Narrative Review of Gene‐Environment Interactions in Pediatric Pulmonology

**DOI:** 10.1002/ppul.71550

**Published:** 2026-03-02

**Authors:** Jelte Kelchtermans

**Affiliations:** ^1^ Children's Hospital of Philadelphia Philadelphia Pennsylvania USA; ^2^ Perelman School of Medicine University of Pennsylvania Philadelphia Pennsylvania USA

**Keywords:** environmental exposures, genetic susceptibility, pediatric lung disease, polygenic risk

## Abstract

Genetic and environmental factors are known to shape the onset, course, and outcomes of pediatric lung disease, yet interactions between these variables are rarely considered in clinical practice. Gene‐environment (GxE) interactions occur when the combined effect of a genetic variant and an environmental exposure differs from what would be expected from combining the effect of either variable alone. Considering these interactions may reveal subgroups at heightened risk, explain apparent inconsistencies across studies, and highlight opportunities for targeted intervention. In this narrative review, we outline the conceptual basis of GxE interactions, including the importance of scale, centering, and timing. We summarize their implications and provide a high‐level overview of available analytic strategies. We then examine disease‐specific findings in asthma, cystic fibrosis, and bronchopulmonary dysplasia, highlighting how GxE processes can shape lung health from early life into adulthood and may contribute to chronic obstructive pulmonary disease risk. Across the asthma and cystic fibrosis examples presented here, commonly reported interactions involve genes related to host defense and oxidative stress biology, with effects that appear sensitive to exposure timing and developmental context. We conclude by identifying near‐term priorities for improving rigor, mechanistic integration, and translation. Together, these insights frame pediatric lung disease within a life‐course GxE model, emphasizing that genetic susceptibility and environmental risk should be considered jointly to improve outcomes.

## 1 Introduction

From asthma to interstitial lung diseases, both environmental and genetic factors shape morbidity and mortality in nearly all conditions managed by pediatric pulmonologists [1, 2, 3]. Yet in clinical practice, only a handful of environmental exposures and genetic variants are directly targeted. On the environmental side, tobacco smoke remains the most established focus, with decades of research devoted to cessation counseling [4, 5, 6]. On the genetic side, therapies directed at specific *cystic fibrosis transmembrane conductance regulator* (*CFTR*) variants have transformed outcomes in cystic fibrosis (CF) [7].

These are powerful but narrow examples, illustrating that translation is possible when the exposure is obvious (tobacco smoke) or the genetic mechanism is well‐defined (CFTR). However, most pediatric lung disease does not arise from a single dominant exposure or a single actionable variant. Children inhale hundreds of thousands of liters of air each day, exposing a vast alveolar surface, thinly separated from the pulmonary circulation and unprotected by hepatic first‐pass metabolism, to a wide array of environmental agents [8]. At the same time, the development of a lung that can efficiently ventilate, accommodate the full cardiac output, and undergo constant immune surveillance requires precise genetic regulation [9]. Together, these observations suggest that more subtle environmental insults and modest genetic risk factors should also have meaningful clinical consequences. Why, then, has clinical translation been so limited?

One explanation is that the respiratory system has substantial physiologic reserve. As a result, many genetic and environmental influences may present not as dramatic short‐term events but as modest shifts in lung growth trajectories that accumulate across the life course. These effects may be difficult to appreciate without longitudinal follow‐up. This creates a statistical challenge since small effect sizes, heterogeneous exposure timing, and imperfect measurement of both exposures and intermediate outcomes, such as lung function or imaging findings, can dilute signal and limit reproducibility. As such, large and well‐phenotyped populations followed longitudinally may be required to estimate effects precisely. Considering gene‐environment (GxE) interactions may provide a way forward. By definition, a GxE interaction occurs when the combined effect of a genetic factor and an environmental exposure deviates from what would be expected based on their independent effects [10]. Importantly, if environmental effects are concentrated in genetically susceptible individuals, analyses that ignore effect modification can dilute effects toward the null by averaging across individuals with heterogeneous susceptibility. In this setting, focusing on high‐risk genetic subgroups can yield larger, more detectable, and potentially more actionable exposure effects than either factor alone.

This narrative review will outline the conceptual basis of GxE interactions, discuss their statistical implications, provide a broad overview of study designs for their investigation, and summarize specific examples in pediatric pulmonology. The goal is to provide a framework for integrating GxE thinking into both research and clinical practice.

## 2 Definitions and Statistical Concepts

### 2.1 Interpreting GxE Interactions

A GxE interaction is present when the effect of an environmental exposure on an outcome differs across genotypes or equivalently, when genetic effects differ by environmental exposure [10]. To interpret any reported GxE effects it is essential to understand the scale used to quantify the effect and how reported variables were centered.

**Figure 1:**
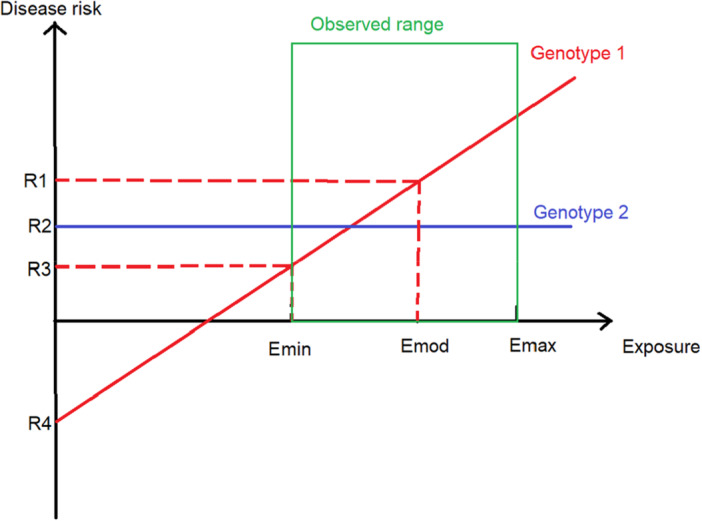
Why centering matters for interpreting GxE effects ‐ Illustrative example. Disease risk (*Y*‐axis; relative risk on the model linear predictor scale) is shown across a hypothetical exposure gradient (*X*‐axis). The green box denotes the empirically observed exposure range within the cohort. The solid red line represents Genotype 1, illustrating exposure‐dependent risk consistent with a positive GxE interaction. The solid blue line represents Genotype 2, illustrating exposure‐independent risk with a flat slope. Vertical markers indicate the lowest observed exposure (Emin), the typical or modal cohort exposure selected as the centering reference (Emod), and the highest observed exposure (Emax). For exposures such as PM_2.5_, these values reflect empirically observed cohort levels (e.g., Emin = 5 µg/m^3^, Emod = 8 µg/m^3^), whereas a value of zero may be unobserved. R1 denotes the predicted risk for Genotype 1 at Emod, R2 denotes the predicted risk for Genotype 2, R3 denotes predicted risks evaluated at Emin, and R4 denotes the extrapolated Genotype 1 prediction when the model is centered at an exposure baseline of zero. Values below zero reflect lower relative risk on the model scale rather than negative clinical risk. The figure illustrates that centering at a clinically meaningful exposure yields genotype main effects interpretable under typical exposure conditions, while the interaction term captures how predicted risk changes as exposure deviates from that reference. [Color figure can be viewed at wileyonlinelibrary.com]

First, GxE interactions can be examined on either the multiplicative or the additive scale (Table 1) [11]. On the multiplicative scale, interaction testing asks whether the exposure produces a different *relative* effect across genotypes (e.g., risk ratio, odds ratio, or hazard ratio). On the additive scale, interaction asks whether the exposure produces a different *absolute* change in risk across genotypes. For example, suppose patients with asthma and genotype A have a 5% annual asthma exacerbation risk and those with genotype B have a 20% risk. If a pollution spike doubles exacerbation risk in both groups, risks become 10% and 40%. In that case, the relative effect is the same (no multiplicative interaction), but the absolute risk increase is larger in genotype B (20 percentage points vs 5), indicating an additive interaction. Conversely, an exposure that produces the same absolute risk increase in both groups would have a different relative effect. Thus, interaction may be present on one scale but not the other, and studies should specify which scale is being used. Second, most GxE regression models include a genetic main effect, an exposure main effect, and an interaction term. By construction, the genetic main effect is defined at a reference exposure level, and the exposure main effect is defined at a reference genotype [12]. This can become problematic when the reference is clinically implausible. For air pollutants, zero exposure is rarely observed. If zero is used as the reference, the reported genetic main effect describes an extrapolated baseline unlikely to be encountered clinically (Figure 1). By contrast, centering continuous exposures at a clinically meaningful value such as the sample mean, a regulatory threshold, or a typical “clean air” day, will provide the genetic effect under usual conditions, with the GxE term capturing how that effect strengthens or weakens as exposure departs from that norm. Taken together, these choices can substantially change how results are expressed and compared across studies. For reproducible interpretation, GxE reports should clearly specify the effect scale and the chosen reference values, and whenever feasible present both relative and absolute contrasts.

**Table 1:** Additive and multiplicative scales of gene‐environment interaction with illustrative examples.

Scale	Question asked	Example scenario
Multiplicative scale (relative)	Does the *relative risk (ratio)* with genotype + exposure differ from the product of independent effects?	*Example 1:* Genotype A baseline = 5% exacerbation risk/year Genotype B baseline = 20% exacerbation risk/year If pollution adds 10% exacerbation risk/year → A = 15%, B = 30% Absolute increases: A + 10 = B + 10 → No interaction on additive scale Relative increases: A x3 ≠ B x1.5 → Interaction on multiplicative scale
Additive scale (absolute)	Does the *absolute excess risk* with genotype + exposure differ from the sum of risks from each alone?	*Example 2:* Genotype A baseline = 5% exacerbation risk/year Genotype B baseline = 20% exacerbation risk/year If pollution doubles risk → A = 10%, B = 40% Absolute increases: A + 5 ≠ B + 20 → Interaction on additive scale Relative increases: A x2 = B x2 → No interaction on multiplicative scale

### 2.2 The Implications of GxE Interactions

GxE interactions matter because exposure effects can differ across genotypes. When this heterogeneity is present, analyses that average across genetically diverse individuals can obscure subgroup‐specific effects, while modeling GxE can clarify which patients are most vulnerable and therefore most likely to benefit from exposure reduction or tailored therapy [13].

This susceptibility stratification also has practical implications for study design. Nissen et al., examined lung function attainment in prematurely born children stratified by polygenic risk for COPD [14, 15]. Those in the highest decile of genetic risk had a 51.4% chance of FEV1 < 5th percentile by age six, compared with 36% in the lowest decile. Because of this difference in baseline risk, a hypothetical trial aimed to reduce this risk by 20% would require a sample size 44% smaller in the high‐risk group than in the low‐risk group to achieve the same statistical power. In other words, considering GxE has the potential to improve statistical efficiency of research by enriching studies for individuals with higher baseline risk or stronger exposure responsiveness, increasing event rates and reducing the sample size needed to have appropriate power to detect a specific effect size. This same concept, that genetic effects can vary across exposure contexts (and vice versa), can also explain apparent inconsistencies across studies. When a GxE interaction is present, the main effect of either factor is not fixed but shifts with exposure context (Figure 1). As a result, a genetic association that appears strong in one population may be attenuated or absent in another if background exposures differ.

In practice, this problem is amplified by exposure measurement limitations. Differences in spatial resolution, timing, or exposure metrics can disproportionately weaken interaction estimates by increasing measurement error and reducing signal‐to‐noise, even when main effects remain detectable. This is particularly important in pediatrics, where developmental windows make timing critical. For example, a genetic variant that increases sensitivity to RSV may have markedly different consequences if infection occurs in early infancy versus later childhood.

Taken together, GxE models are valuable not only for etiologic insight, but also because they can reveal where risk is concentrated and help prioritize the patients and exposure contexts in which interventions are most likely to yield meaningful clinical benefit [16].

### 2.3 Statistical Approaches to GxE

The next question is how to test for GxE in practice. GxE analyses are constrained by the high multiple‐testing burden of genome‐wide studies and by reduced signal‐to‐noise due to measurement error in real‐world exposure assessment [17]. Together, these issues can substantially limit power for detecting interactions, even in large cohorts. As a result, investigators typically choose between approaches designed for targeted, interpretable interaction testing versus approaches optimized for genome‐wide discovery, often with different trade‐offs between interpretability and sensitivity (Table 2) [17].

**Table 2:** Summary of widely used GxE analytic strategies, highlighting the primary advantage and key limitation of each approach.

Approach	Advantages	Limitations
Regression GxE term (single variant)	Simple; interpretable	Low power; sensitive to exposure error + rare variants/exposures
Genome‐wide GxE scan	Unbiased discovery	Huge multiple‐testing burden; limited power
Joint test (G + GxE)	More sensitive than GxE‐only	Harder to separate main vs interaction signal
Two‐stage screening (GxE after filtering)	Reduces testing burden	Will miss interactions not captured by screen
Polygenic/pathway score x exposure	Higher power; clinically aligned	Depends on source data; low mechanistic resolution
Case‐only design	High sensitivity if assumptions hold	Bias if genotype‐exposure independence violated
Family‐based (trios)	Robust to population stratification	Limited availability
Multi‐omic integration	Supports mechanistic interpretation	Complex; needs large samples + relevant tissue

In candidate interaction studies, the most straightforward strategy is to include an interaction term in a regression model. In linear, logistic, or Cox regression, adding a GxE product term directly tests whether the exposure effect differs by genotype. This approach is transparent and clinically interpretable, but it is often underpowered, particularly when exposures are uncommon or measured imprecisely, variant frequencies are low, or outcomes are rare [16, 17].

In genome‐wide settings, testing a separate GxE term for millions of variants rapidly loses statistical power [16]. To improve discovery efficiency, several strategies have been developed. First, joint tests combine evidence from the genetic main effect and the GxE term into a single statistic, improving sensitivity across a broader range of underlying models [16]. Second, two‐stage approaches prioritize variants for interaction testing based on marginal genetic association or biological annotation, reducing the multiple‐testing burden at the cost of likely missing some true GxE effects [17]. Third, dimension‐reduction approaches can aggregate genetic information, such as polygenic risk score (PRS) x exposure models, to improve power and biological interpretability. These approaches can be more aligned with risk stratification, but their performance depends on consistent underlying effect directionality and on the quality and ancestry match of the underlying genome‐wide association study [12, 16, 17].

Beyond analytic strategy, study design can also improve efficiency under specific assumptions. Case‐only designs can be attractive because, if genotype and exposure are independent in the source population, over‐representation of a genotype among cases with higher exposure (e.g., asthma exacerbations after high‐pollution days) can provide evidence of GxE. The key limitation is that violations of genotype‐exposure independence can create spurious interactions, which is a concern in pediatrics due to correlations between exposure, place, ancestry, and socioeconomic conditions [18]. Additionally, family‐based designs, such as case‐parent trios, compare transmitted and non‐transmitted alleles within families, limiting confounding from population structure, but are less commonly feasible outside specialized cohorts [18].

Finally, multi‐omic approaches that integrate genetic data with epigenetic or transcriptomic variation can help connect statistical interactions to plausible mechanisms. These approaches can be informative, but they typically require careful design, large sample sizes, and substantial computational infrastructure, and they raise additional challenges related to confounding, tissue specificity, and technical variation [19].

Taken together, no single method solves all challenges in GxE analysis. Candidate interaction studies often prioritize transparent regression‐based models, while genome‐wide discovery typically requires strategies that address multiple testing and limited power. Across approaches, clear reporting of the effect scale, coding and centering decisions, and stratum‐specific estimates remains essential for reproducible interpretation.

## 3 Known GxE Interactions in Pediatric Pulmonology

### 3.1 Asthma

Asthma is the pediatric lung disease where GxE interactions have been studied most extensively, perhaps because of the inherent heterogeneity in both disease presentation and progression [20, 21, 22, 23, 24, 25, 26]. Although many interactions have been proposed, several recurring themes have emerged across the literature.

First, is that timing appears to be an important variable. Exposures exert very different effects depending on whether they occur in utero, during infancy, or later in childhood [27, 28]. Similarly, the effect of genetic variants is not static, with risk alleles magnifying vulnerability to pollutants or allergens during critical developmental windows but carrying little weight outside those periods [27]. For example, the increased risk for asthma related to variants in the 17q21 region as initially described was increased, on a multiplicative scale, by early life exposure to environmental tobacco smoke [29]. Prior exposures also influence gene expression and methylation patterns, altering responses to later environmental stimuli [20, 27, 30]. Thus, variation in exposure timing can introduce substantial noise, biasing studies toward null results and complicating replication.

A second theme is the concentration of research on a limited set of exposures. Environmental tobacco smoke was among the earliest studied, with consistent findings that variants in detoxification and immune pathways modify both prenatal and postnatal effects on asthma [31, 32, 33, 34]. Air pollution exposure has seen a similar early interest in pediatric asthma GxE studies with genetic variation in detoxification genes first demonstrated to modify the impact of air pollution exposure in the 2000's [35, 36]. Perhaps unsurprisingly, genetic variants that modify the effect of air pollution described to date again appear to be centered on detoxification and immune pathways [37]. Farming and microbial exposures have also drawn attention, particularly in relation to the hygiene hypothesis [20, 25, 28]. Pharmacogenetic studies extend the concept further, demonstrating that variants in ADRB2, FCER2, and related pathways influencing the response to inhaled corticosteroids and β‐agonists, often in ways that vary by environmental context [38, 39].

Despite heterogeneity, a few signals have been reported across multiple studies. The interactions between variants on the 17q21 locus and early viral infections is the best replicated finding in asthma genetics [20, 34]. Furthermore, genes in the glutathione pathway consistently appear to modify responses to tobacco smoke and air pollution, despite ongoing heterogeneity in published findings [40]. Furthermore, CD14 and related innate immunity genes interact with endotoxin or microbial load, though the direction of effect depends on timing and exposure context [22]. These recurrent signals contrast with the broader set of candidate‐gene findings that remain unreplicated, likely reflecting methodological heterogeneity and the historical reliance on candidate approaches, with genome‐wide and polygenic interaction studies only recently entering the pediatric asthma literature [41, 42, 43, 44].

Furthermore, epigenetic work has extended the GxE paradigm beyond DNA sequence variation, as comprehensively reviewed by Hernandez‐Pacheco et al, with epigenetic studies demonstrating that DNA methylation and other marks can mediate exposure effects and, in turn, modify susceptibility [20, 27, 30]. This work provides a mechanistic bridge linking early exposures to long‐term disease trajectories.

Taken together, the asthma GxE literature highlights that exposure timing and measurement remain key challenges. Reported interactions often depend on when exposures occur and how precisely they are captured, which can introduce heterogeneity across studies and complicate replication. These observations reinforce that future asthma GxE studies will benefit from improved exposure assessment and careful attention to developmental windows.

### 3.2 Cystic Fibrosis

Although CF arises from mutations in a single gene, disease expression varies widely [45]. Even among patients carrying identical Cystic Fibrosis Transmembrane Conductance Regulator (CFTR) variants, lung function, infection burden, and survival can diverge dramatically, underscoring that CFTR genotype alone does not explain severity [45]. CF therefore illustrates how, even in a monogenic disease, both genetic modifiers and environmental exposures interact to shape outcomes.

One of the clearest GxE findings in CF involves secondhand smoke. *Collaco* et al. demonstrated that children carrying risk alleles in TGFB1 experience steeper declines in lung function when exposed to tobacco smoke [46]. A second well‐documented case is *MBL2*, where low‐expression variants increase vulnerability to chronic infection [47]. Patients with these variants who acquire *Pseudomonas aeruginosa* experience more rapid pulmonary decline, while differences are muted in those who remain uninfected [47, 48, 49]. Oxidative stress pathways have also been implicated with early studies linking deletions in *GSTM1* as risk factors for worse pulmonary outcomes [50]. Marson et al later confirmed that polymorphisms in glutathione metabolism genes worsen lung disease in patients exposed to higher oxidative burdens [51]. However, to our knowledge, this work has not yet been extended to explore a potential interaction with air pollution as extensively studied in pediatric asthma [40].

The advent of CFTR modulators has brought GxE interactions into renewed focus. Early evidence suggests that both environmental exposures and genetic modifiers may influence the effectiveness of these transformative therapies [52]. Optimizing outcomes will therefore require research frameworks that integrate CFTR genotype, modifier genes, and environmental exposures simultaneously, considering both main effects and interactions. Such an approach may help ensure that the benefits of CFTR modulators are realized equitably across the CF population.

### 3.3 Lifelong GxE Effects: From BPD to COPD

The developing lung provides a unique window into how GxE interactions influence pulmonary health across the lifespan. Children born prematurely are exposed to distinctive neonatal stressors, including hyperoxia, mechanical ventilation, and early infection, that interact with genetic background to determine the risk of bronchopulmonary dysplasia (BPD) [53]. BPD reflects an imbalance between lung growth, injury, and repair, defined clinically by the need for supplemental oxygen or respiratory support near term [53]. Although more than half of infants with BPD discharged with oxygen can be weaned to room air within a year, their reduced pulmonary reserve may leave them uniquely sensitive to later environmental insults, illustrating how early GxE interactions set the stage for long‐term vulnerability [54].

This early vulnerability carries lifelong consequences. Children with BPD experience higher hospitalization rates, reduced lung growth trajectories, and in many cases reach an early plateau in lung function, increasing their risk of developing chronic obstructive pulmonary disease (COPD) in adulthood [53, 55, 56, 57, 58]. Yet outcomes vary markedly even among preterm infants with similar exposures, highlighting the role of genetic variation and potential GxE interactions in shaping disease course [59, 60, 61, 62].

Prior studies support the notion that genetic variation contributes to this variability. In a large phenotype‐wide association study (PheWAS), our group found that the IL18R1/IL18RAP locus not only modified BPD risk but also influenced lung function attainment as well as risk for asthma and COPD, underscoring that genetic effects can manifest differently depending on developmental stage and exposure context [63]. Polygenic approaches extend this principle. Moll et al. developed a PRS for COPD from > 1.7 million spirometry‐associated variants in adults [14]. Although derived from adult cohorts, this PRS also predicted impaired lung function in children with asthma and in preterm infants, particularly those exposed to neonatal respiratory stress [14, 64, 65]. In other words, the same genetic background may impair lung growth in infancy when combined with hyperoxia or mechanical ventilation yet contribute to accelerated lung function decline in adulthood when paired with cigarette smoke or air pollution. These findings speak to genetic risk expressing differently across clinical contexts more than they provide definitive GxE interaction tests but underscore the need for studies that explicitly model how neonatal and later‐life exposures modify genetic susceptibility.

Taken together, these examples support the notion of a life‐course GxE framework. While an individual's inherited genetic background is fixed, its clinical consequences are context‐dependent, shifting across age‐specific exposures such as oxygen therapy in infancy, viral or allergen exposures in childhood, and smoking or air pollution in adulthood. Recognizing this continuity underscores the need for prevention and intervention strategies that integrate genetic susceptibility with evolving environmental risks across the lifespan.

## 4 Conclusion and Future Directions

The studies reviewed here underscore that pediatric lung disease is shaped not by genetics or environment alone, but by their interaction across development. Across asthma, cystic fibrosis, BPD, and COPD, GxE effects can contribute to risk and clinical heterogeneity. Importantly, these effects appear highly dependent on developmental context, with susceptibility expressed differently across exposures and time.

Several issues will need to be addressed to move the field forward. First, because interaction estimates depend on choices like analytic scale, centering, and model specification, studies should report these decisions clearly and present results in ways that are clinically interpretable, including both absolute and relative effects at relevant exposure levels. Second, progress will increasingly depend on mechanistic integration. Pairing genetic data with high‐resolution exposure measurement and functional genomics may help identify critical windows of vulnerability and link interaction signals to biologic pathways. Third, translation needs to extend beyond discovery. If GxE studies identify subgroups at elevated risk, interventions should be designed with those subgroups in mind, whether reducing smoke exposure in genetically susceptible children with asthma, tailoring oxygen strategies in preterm infants at highest polygenic risk, or accounting for modifier‐infection combinations in cystic fibrosis.

Taken together, pediatric pulmonology fits naturally within a life‐course GxE model. The same genetic background may predispose to BPD in the NICU, asthma in childhood, or COPD in adulthood, depending on the exposures encountered. Recognizing this continuity reframes pediatric lung disease not only as a matter of acute care, but also as a prevention problem across the lifespan.

## Author Contributions

**Jelte Kelchtermans:** conceptualization, writing – original draft, data curation, visualization.

## AI Disclosure

The authors used ChatGPT (OpenAI) to support language refinement. The authors reviewed and take full responsibility for all content.

## Conflicts of Interest

The author declares no conflicts of interest.

## Data Availability

Data sharing not applicable to this article as no datasets were generated or analysed during the current study.

## References

[1] M. H. Hawley , P. P. Moschovis , M. Lu , T. B. Kinane , and L. M. Yonker , “The Future Is Here: Integrating Genetics Into the Pediatric Pulmonary Clinic,” Pediatric Pulmonology 55, no. 7 (2020): 1810–1818. 32533912 10.1002/ppul.24723 PMC7384239

[2] A. Hamvas , B. P. Chaudhari , and L. M. Nogee , “Genetic Testing for Diffuse Lung Diseases in Children,” Pediatric Pulmonology 59, no. 9 (2024): 2286–2297. 37191361 10.1002/ppul.26447

[3] P. D. Sly , “Adverse Environmental Exposure and Respiratory Health in Children,” Pediatric Clinics of North America 68, no. 1 (2021): 277–291. 33228938 10.1016/j.pcl.2020.09.018

[4] E. W. Kligman and S. Narce‐Valente , “Reducing the Exposure of Children to Environmental Tobacco Smoke. An Office‐Based Intervention Program,” Journal of Family Practice 30, no. 3 (1990): 263–269. 2307940

[5] T. G. Irons , S. T. White , and R. D. Kenney , “Parental Smoking Cessation Counseling by Pediatric Residents,” Pediatric Research 21, no. 4 (1987): 257A.3562124

[6] E. M. Mahabee‐Gittens , R. T. Ammerman , J. C. Khoury , et al., “A Parental Smoking Cessation Intervention in the Pediatric Emergency Setting: A Randomized Trial,” International Journal of Environmental Research and Public Health 17, no. 21 (2020): 8151. 33158230 10.3390/ijerph17218151 PMC7663571

[7] J. L. Taylor‐Cousar , P. D. Robinson , M. Shteinberg , and D. G. Downey , “CFTR Modulator Therapy: Transforming the Landscape of Clinical Care in Cystic Fibrosis,” Lancet 402, no. 10408 (2023): 1171–1184. 37699418 10.1016/S0140-6736(23)01609-4

[8] J. D. Pleil , M. Ariel Geer Wallace , M. D. Davis , and C. M. Matty , “The Physics of Human Breathing: Flow, Timing, Volume, and Pressure Parameters for Normal, On‐Demand, and Ventilator Respiration,” Journal of Breath Research 15, no. 4 (2021): 042002. 10.1088/1752-7163/ac2589 PMC8672270 34507310

[9] M. Z. Nikolić , D. Sun , and E. L. Rawlins , “Human Lung Development: Recent Progress and New Challenges,” Development 145, no. 16 (2018): dev163485. 30111617 10.1242/dev.163485 PMC6124546

[10] K. J. Rothman , S. Greenland , and A. M. Walker , “Concepts of Interaction,” American Journal of Epidemiology 112, no. 4 (1980): 467–470. 7424895 10.1093/oxfordjournals.aje.a113015

[11] L. P. Zhao , W. Fan , G. Goodman , J. Radich , and P. Martin , “Deciphering Genome Environment Wide Interactions Using Exposed Subjects Only,” Genetic Epidemiology 39, no. 5 (2015): 334–346. 25694100 10.1002/gepi.21890 PMC4469559

[12] H. Aschard , “A Perspective on Interaction Effects in Genetic Association Studies,” Genetic Epidemiology 40, no. 8 (2016): 678–688. 27390122 10.1002/gepi.21989 PMC5132101

[13] K. McAllister , L. E. Mechanic , C. Amos , et al., “Current Challenges and New Opportunities for Gene‐Environment Interaction Studies of Complex Diseases,” American Journal of Epidemiology 186, no. 7 (2017): 753–761. 28978193 10.1093/aje/kwx227 PMC5860428

[14] M. Moll , P. Sakornsakolpat , N. Shrine , et al., “Chronic Obstructive Pulmonary Disease and Related Phenotypes: Polygenic Risk Scores in Population‐Based and Case‐Control Cohorts,” Lancet Respiratory Medicine 8, no. 7 (2020): 696–708. 32649918 10.1016/S2213-2600(20)30101-6 PMC7429152

[15] G. Nissen , S. Hinsenbrock , T. K. Rausch , et al., “Lung Function of Preterm Children Parsed by a Polygenic Risk Score for Adult COPD,” NEJM Evidence 2, no. 3 (2023): EVIDoa2200279. 38320054 10.1056/EVIDoa2200279

[16] P. Kraft , Y. C. Yen , D. O. Stram , J. Morrison , and W. J. Gauderman , “Exploiting Gene‐Environment Interaction to Detect Genetic Associations,” Human Heredity 63, no. 2 (2007): 111–119. 17283440 10.1159/000099183

[17] M. Wu , Y. Li , and S. Ma , “High‐Dimensional Gene–Environment Interaction Analysis,” Annual Review of Statistics and its Application 12, no. 1 (2025): 361–383. 10.1146/annurev-statistics-112723-034315 PMC12383825 40881670

[18] J. Miao , Y. Wu , and Q. Lu , “Statistical Methods for Gene‐Environment Interaction Analysis,” WIREs Computational Statistics 16, no. 1 (2024): e1635. 38699459 10.1002/wics.1635 PMC11064894

[19] X. Yang , “Multitissue Multiomics Systems Biology to Dissect Complex Diseases,” Trends in Molecular Medicine 26, no. 8 (2020): 718–728. 32439301 10.1016/j.molmed.2020.04.006 PMC7395877

[20] N. Hernandez‐Pacheco , M. Kere , and E. Melén , “Gene‐Environment Interactions in Childhood Asthma Revisited; Expanding the Interaction Concept,” Pediatric Allergy and Immunology 33, no. 5 (2022): e13780. 35616899 10.1111/pai.13780 PMC9325482

[21] H. Johansson , T. B. Mersha , E. B. Brandt , and G. K. Khurana Hershey , “Interactions Between Environmental Pollutants and Genetic Susceptibility in Asthma Risk,” Current Opinion in Immunology 60 (2019): 156–162. 31470287 10.1016/j.coi.2019.07.010 PMC6800636

[22] E. Morales and D. Duffy , “Genetics and Gene‐Environment Interactions in Childhood and Adult Onset Asthma,” Frontiers in Pediatrics 7 (2019): 499. 31921716 10.3389/fped.2019.00499 PMC6918916

[23] D. S. Postma and S. T. Weiss , Gene‐Environment Interaction in Asthma. (Informa Healthcare, 2006), 63–84.

[24] L. Rigoli , S. Briuglia , S. Caimmi , et al., “Gene‐Environment Interaction in Childhood Asthma,” International Journal of Immunopathology and Pharmacology 24, no. 4_suppl (2011): 41–47. 22032786 10.1177/03946320110240S409

[25] C. Sengler , S. Lau , U. Wahn , and R. Nickel , “Interactions Between Genes and Environmental Factors in Asthma and Atopy: New Developments,” Respiratory Research 3, no. 1 (2001): 7. 11806842 10.1186/rr179 PMC64818

[26] D. Vercelli , “Gene–Environment Interactions in Asthma and Allergy: The End of the Beginning?,” Current Opinion in Allergy and Clinical Immunology 10, no. 2 (2010): 145–148. 20051845 10.1097/ACI.0b013e32833653d7 PMC2854841

[27] G. Chatziparasidis , M. R. Chatziparasidi , A. Kantar , and A. Bush , “Time‐Dependent Gene–Environment Interactions Are Essential Drivers of Asthma Initiation and Persistence,” Pediatric Pulmonology 59, no. 5 (2024): 1143–1152. 38380964 10.1002/ppul.26935

[28] E. Morales and D. Duffy , “Genetics and Gene‐Environment Interactions in Childhood and Adult Onset Asthma,” Frontiers in Pediatrics 7 (2019): 499. 31921716 10.3389/fped.2019.00499 PMC6918916

[29] E. Bouzigon , E. Corda , H. Aschard , et al., “Effect of 17q21 Variants and Smoking Exposure in Early‐Onset Asthma,” New England Journal of Medicine 359, no. 19 (2008): 1985–1994. 18923164 10.1056/NEJMoa0806604

[30] H. Harb , B. Alashkar Alhamwe , H. Garn , H. Renz , and D. P. Potaczek , “Recent Developments in Epigenetics of Pediatric Asthma,” Current Opinion in Pediatrics 28, no. 6 (2016): 754–763. 27662207 10.1097/MOP.0000000000000424

[31] S. Colilla , D. Nicolae , A. Pluzhnikov , et al., “Evidence for Gene‐Environment Interactions in a Linkage Study of Asthma and Smoking Exposure,” Journal of Allergy and Clinical Immunology 111, no. 4 (2003): 840–846. 12704367 10.1067/mai.2003.170

[32] D. A. Meyers , D. S. Postma , O. C. Stine , et al., “Genome Screen for Asthma and Bronchial Hyperresponsiveness: Interactions With Passive Smoke Exposure,” Journal of Allergy and Clinical Immunology 115, no. 6 (2005): 1169–1175. 15940130 10.1016/j.jaci.2005.01.070

[33] M. H. Dizier , E. Bouzigon , M. Guilloud‐Bataille , et al., “Evidence for Gene × Smoking Exposure Interactions in a Genome‐Wide Linkage Screen of Asthma and Bronchial Hyper‐Responsiveness in EGEA Families,” European Journal of Human Genetics 15, no. 7 (2007): 810–815. 17426724 10.1038/sj.ejhg.5201830

[34] E. Von Mutius , “Gene‐Environment Interactions in Asthma,” Journal of Allergy and Clinical Immunology 123, no. 1 (2009): 3–11. 19130922 10.1016/j.jaci.2008.10.046

[35] E. Bergamaschi , G. De Palma , P. Mozzoni , et al., “Polymorphism of Quinone‐Metabolizing Enzymes and Susceptibility to Ozone‐Induced Acute Effects,” American Journal of Respiratory and Critical Care Medicine 163, no. 6 (2001): 1426–1431. 11371413 10.1164/ajrccm.163.6.2006056

[36] G. L. David , I. Romieu , J. J. Sienra‐Monge , et al., “Nicotinamide Adenine Dinucleotide (Phosphate) Reduced:Quinone Oxidoreductase and Glutathione S‐Transferase M1 Polymorphisms and Childhood Asthma,” American Journal of Respiratory and Critical Care Medicine 168, no. 10 (2003): 1199–1204. 12969868 10.1164/rccm.200305-684OC

[37] J. Kelchtermans and H. Hakonarson , “The Role of Gene–Ambient Air Pollution Interactions in Paediatric Asthma,” European Respiratory Review 31, no. 166 (2022): 220094. 36384702 10.1183/16000617.0094-2022 PMC9724879

[38] J. Perez‐Garcia , A. Espuela‐Ortiz , F. Lorenzo‐Diaz , and M. Pino‐Yanes , “Pharmacogenetics of Pediatric Asthma: Current Perspectives,” Pharmacogenomics and Personalized Medicine 13 (2020): 89–103. 32256100 10.2147/PGPM.S201276 PMC7090194

[39] H. Duong‐Thi‐Ly , H. Nguyen‐Thi‐Thu , L. Nguyen‐Hoang , H. Nguyen‐Thi‐Bich , T. J. Craig , and S. Duong‐Quy , “Effects of Genetic Factors to Inhaled Corticosteroid Response in Children With Asthma: A Literature Review,” Journal of International Medical Research 45, no. 6 (2017): 1818–1830. 29251255 10.1177/0300060516683877 PMC5805193

[40] X. Dai , D. S. Bui , and C. Lodge , “Glutathione S‐Transferase Gene Associations and Gene‐Environment Interactions for Asthma,” Current Allergy and Asthma Reports 21, no. 5 (2021): 31. 33970355 10.1007/s11882-021-01005-y

[41] P. E. Sugier , C. Sarnowski , R. Granell , et al., “Genome‐Wide Interaction Study of Early‐Life Smoking Exposure on Time‐to‐Asthma Onset in Childhood,” Clinical & Experimental Allergy 49, no. 10 (2019): 1342–1351. 31379025 10.1111/cea.13476

[42] K. Voorhies , J. E. Sordillo , M. McGeachie , et al., “Age by Single Nucleotide Polymorphism Interactions on Bronchodilator Response in Asthmatics,” Journal of Personalized Medicine 11, no. 1 (2021): 59. 33477890 10.3390/jpm11010059 PMC7833432

[43] A. Dahlin , J. E. Sordillo , M. McGeachie , et al., “Genome‐Wide Interaction Study Reveals Age‐Dependent Determinants of Responsiveness to Inhaled Corticosteroids in Individuals With Asthma,” PLoS One 15, no. 3 (2020): e0229241. 32119686 10.1371/journal.pone.0229241 PMC7051058

[44] J. Kelchtermans , M. E. March , F. Mentch , et al., “Genetic Modifiers of Asthma Response to Air Pollution in Children: An African Ancestry GWAS and PM(2.5) Polygenic Risk Score Study,” Environmental Research 267 (2025): 120666. 39725137 10.1016/j.envres.2024.120666 PMC11800831

[45] M. A. Mall , P.‐R. Burgel , C. Castellani , J. C. Davies , M. Salathe , and J. L. Taylor‐Cousar , “Cystic Fibrosis,” Nature Reviews Disease Primers 10, no. 1 (2024): 53. 10.1038/s41572-024-00538-6 39117676

[46] J. M. Collaco , L. Vanscoy , L. Bremer , et al., “Interactions Between Secondhand Smoke and Genes That Affect Cystic Fibrosis Lung Disease,” Journal of the American Medical Association 299, no. 4 (2008): 417–424, 10.1001/jama.299.4.417. 18230779 PMC3139475

[47] J. M. Collaco and G. R. Cutting , “Update on Gene Modifiers in Cystic Fibrosis,” Current Opinion in Pulmonary Medicine 14, no. 6 (2008): 559–566. 18812833 10.1097/MCP.0b013e3283121cdc PMC2785460

[48] C. Trevisiol , M. Boniotto , L. Giglio , F. Poli , M. Morgutti , and S. Crovella , “MBL2 Polymorphisms Screening in a Regional Italian CF Center,” Journal of Cystic Fibrosis 4, no. 3 (2005): 189–191. 16046196 10.1016/j.jcf.2005.04.001

[49] K. E. McDougal , D. M. Green , L. L. Vanscoy , et al., “Use of a Modeling Framework to Evaluate the Effect of a Modifier Gene (MBL2) on Variation in Cystic Fibrosis,” European Journal of Human Genetics 18, no. 6 (2010): 680–684. 20068595 10.1038/ejhg.2009.226 PMC2874654

[50] J. Hull and A. H. Thomson , “Contribution of Genetic Factors Other Than CFTR to Disease Severity in Cystic Fibrosis,” Thorax 53, no. 12 (1998): 1018–1021. 10195071 10.1136/thx.53.12.1018 PMC1745152

[51] F. A. L. Marson , C. S. Bertuzzo , R. Secolin , A. F. Ribeiro , and J. D. Ribeiro , “Genetic Interaction of GSH Metabolic Pathway Genes in Cystic Fibrosis,” BMC Medical Genetics 14, no. 1 (2013): 60. 23758905 10.1186/1471-2350-14-60 PMC3685592

[52] A. Sepahzad , D. J. Morris‐Rosendahl , and J. C. Davies , “Cystic Fibrosis Lung Disease Modifiers and Their Relevance in the New Era of Precision Medicine,” Genes 12, no. 4 (2021): 562. 33924524 10.3390/genes12040562 PMC8069009

[53] B. Thébaud , K. N. Goss , M. Laughon , et al., “Bronchopulmonary Dysplasia,” Nature Reviews Disease Primers 5, no. 1 (2019): 78. 10.1038/s41572-019-0127-7 PMC6986462 31727986

[54] J. Yeh , S. A. McGrath‐Morrow , and J. M. Collaco , “Oxygen Weaning After Hospital Discharge in Children With Bronchopulmonary Dysplasia,” Pediatric Pulmonology 51, no. 11 (2016): 1206–1211. 27093064 10.1002/ppul.23442 PMC5556369

[55] C. Siffel , K. D. Kistler , J. F. M. Lewis , and S. P. Sarda , “Global Incidence of Bronchopulmonary Dysplasia Among Extremely Preterm Infants: A Systematic Literature Review,” Journal of Maternal‐Fetal & Neonatal Medicine: The Official Journal of the European Association of Perinatal Medicine, the Federation of Asia and Oceania Perinatal Societies, the International Society of Perinatal Obstetricians 34, no. 11 (2021): 1721–1731. 10.1080/14767058.2019.1646240 31397199

[56] J. P. Kinsella , A. Greenough , and S. H. Abman , “Bronchopulmonary Dysplasia,” Lancet 367, no. 9520 (2006): 1421–1431. 16650652 10.1016/S0140-6736(06)68615-7

[57] W. Lapcharoensap , M. V. Bennett , X. Xu , H. C. Lee , and D. Dukhovny , “Hospitalization Costs Associated With Bronchopulmonary Dysplasia in the First Year of Life,” Journal of Perinatology 40, no. 1 (2020): 130–137. 31700090 10.1038/s41372-019-0548-x PMC6920537

[58] J. M. Collaco and S. A. McGrath‐Morrow , “Respiratory Phenotypes for Preterm Infants, Children, and Adults: Bronchopulmonary Dysplasia and More,” Annals of the American Thoracic Society 15, no. 5 (2018): 530–538. 29328889 10.1513/AnnalsATS.201709-756FR

[59] K.‐H. Yu , J. Li , M. Snyder , G. M. Shaw , and H. M. O'Brodovich , “The Genetic Predisposition to Bronchopulmonary Dysplasia,” Current Opinion in Pediatrics 28, no. 3 (2016): 318–323. 26963946 10.1097/MOP.0000000000000344 PMC4853271

[60] A. Hadchouel , X. Durrmeyer , E. Bouzigon , et al., “Identification of SPOCK2 as a Susceptibility Gene for Bronchopulmonary Dysplasia,” American Journal of Respiratory and Critical Care Medicine 184, no. 10 (2011): 1164–1170. 21836138 10.1164/rccm.201103-0548OC PMC4826668

[61] J. Li , K. H. Yu , J. Oehlert , et al., “Exome Sequencing of Neonatal Blood Spots and the Identification of Genes Implicated in Bronchopulmonary Dysplasia,” American Journal of Respiratory and Critical Care Medicine 192, no. 5 (2015): 589–596. 26030808 10.1164/rccm.201501-0168OC PMC4595691

[62] F. Blume , H. Kirsten , P. Ahnert , et al., “Verification of Immunology‐Related Genetic Associations in BPD Supports ABCA3 and Five Other Genes,” Pediatric Research 92, no. 1 (2022): 190–198. 34465876 10.1038/s41390-021-01689-y PMC9411063

[63] J. Kelchtermans , M. E. March , H. Hakonarson , and S. A. McGrath‐Morrow , “Phenotype Wide Association Study Links Bronchopulmonary Dysplasia With Eosinophilia in Children,” Scientific Reports 14, no. 1 (2024): 21391. 39271728 10.1038/s41598-024-72348-5 PMC11399246

[64] G. Nissen , S. Hinsenbrock , T. K. Rausch , et al., “Lung Function of Preterm Children Parsed by a Polygenic Risk Score for Adult COPD,” NEJM Evidence 2, no. 3 (2023): EVIDoa2200279. 38320054 10.1056/EVIDoa2200279

[65] M. J. McGeachie , K. P. Yates , X. Zhou , et al., “Patterns of Growth and Decline in Lung Function in Persistent Childhood Asthma,” New England Journal of Medicine 374, no. 19 (2016): 1842–1852. 27168434 10.1056/NEJMoa1513737 PMC5032024

